# Crosstalk between MicroRNAs and Peroxisome Proliferator-Activated Receptors and Their Emerging Regulatory Roles in Cardiovascular Pathophysiology

**DOI:** 10.1155/2018/8530371

**Published:** 2018-12-05

**Authors:** Yin-Feng Zhang, Hai-Ming Xu, Fei Yu, Man Wang, Meng-Yang Li, Tao Xu, Yan-Yan Gao, Jian-Xun Wang, Pei-Feng Li

**Affiliations:** ^1^Institute for Translational Medicine, College of Medicine, Qingdao University, Deng Zhou Road 38, Qingdao 266021, China; ^2^Department of Occupational and Environmental Medicine, School of Public Health and Management, Ningxia Medical University, Yinchuan, Ningxia Hui Autonomous Region, China

## Abstract

Peroxisome proliferator-activated receptors (PPARs) play vital roles in cardiovascular pathophysiology, such as energy balance, cell proliferation/apoptosis, inflammatory response, and adipocyte differentiation. These vital roles make PPARs potential targets for therapeutic prevention of cardiovascular diseases (CVDs). Emerging evidence indicates that the crosstalk of microRNAs (miRNAs) and PPARs contributes greatly to CVD pathogenesis. PPARs are inhibited by miRNAs at posttranscriptional mechanisms in the progress of pulmonary hypertension and vascular dysfunction involving cell proliferation/apoptosis, communication, and normal function of endothelial cells and vascular smooth muscle cells. In the development of atherosclerosis and stroke, the activation of PPARs could change the transcripts of target miRNA through miRNA signalling. Furthermore, the mutual regulation of PPARs and miRNAs involves cell proliferation/apoptosis, cardiac remodeling, and dysfunction in heart diseases. In addition, obesity, an important cardiovascular risk, is modulated by the regulatory axis of PPARs/miRNAs, including adipogenesis, adipocyte dysfunction, insulin resistance, and macrophage polarization in adipose tissue. In this review, the crosstalk of PPARs and miRNAs and their emerging regulatory roles are summarized in the context of CVDs and risks. This provides an understanding of the underlying mechanism of the biological process related to CVD pathophysiology involving the interaction of PPARs and miRNAs and will lead to the development of PPARs/miRNAs as effective anti-CVD medications.

## 1. Introduction

Peroxisome proliferator-activated receptors (PPARs) belong to the family of ligand-activated nuclear hormone receptors, and they serve as important regulators in many physiological processes, including modulation of the metabolism of carbohydrates, lipids, and proteins; cellular proliferation and differentiation; inflammation; and tumorigenesis [1]. PPARs heterodimerize with the retinoid X receptor and subsequently bind to peroxisome proliferator response elements (PPREs) in the DNA of target genes [2]. PPARs include three isoforms—PPAR*α*, PPAR*β*/*δ*, and PPAR-*γ*—that present many different features such as ligand specificities, tissue distribution, coactivators or corepressors specificities, and effects [3]. Emerging evidence has demonstrated that PPARs have a wide range of biological activities that prevent and treat CVDs [4]. Moreover, the availability of natural and synthetic small molecule agonists, many of which are relatively well-studied, makes PPARs attractive therapeutic targets [5, 6].

### 1.1. Cardiovascular Diseases (CVDs)

Cardiovascular diseases (CVDs) are the leading cause of death all over the world. Atherosclerosis (ATH), hypertension, myocardial infarction, and cerebrovascular disease (stroke) are the most common CVDs that involve the heart and blood vessels [7–9]. Heart failure (HF) causes the most deaths worldwide and is usually associated with cardiac hypertrophy and cardiomyocyte apoptosis [10]. Obesity, a worldwide epidemic, is an important risk factor for CVDs [11]. This inflammatory condition is caused by both adipocyte hypertrophy and adipogenesis.

ATH is a complicated vascular disease that can be ascribed to many factors [7, 12]. In the development of ATH, the proliferation of vascular smooth muscle cells (VSMCs) and damage to endothelial cell (ECs) resulting in the expression of adhesion molecules and leukocyte adhesion are important events [13, 14]. Additionally, accumulation of low-density lipoproteins, monocytes, and macrophages constitutes a plaque in the vascular wall. When the atherosclerotic plaque builds and becomes fragile, it can rupture, causing a variety of leading death diseases, such as stroke and myocardial infarction [15].

Pulmonary hypertension (PH) is an enigmatic vascular disorder driven by disparate triggers such as inflammation and hypoxia, and it results in significant morbidity and mortality [16]. Development of PH involves various molecular pathways that include several cell types especially pulmonary arterial smooth muscle cells (PASMCs) and pulmonary arterial ECs (PAECs) [17].

### 1.2. Role of Peroxisome Proliferator-Activated Receptors (PPARs) in CVDs

#### 1.2.1. PPAR*α*

The first cloned PPAR isotype was PPAR*α*, which is expressed predominantly in high-energy requiring tissue such as brown adipose tissue and the parenchymal cells of the heart [18–20]. The beneficial effect of PPAR*α* in the pathogenesis of ATH and management of cardiomyocyte metabolism has been studied extensively [21, 22].

Activation of PPAR*α* has been demonstrated to raise the plasma levels of atheroprotective high-density lipoprotein cholesterol and reduce circulating levels of triglycerides (TG), free fatty acids (FAs), and apolipoprotein CIII. PPAR*α* activation has also been shown to improve the overall proatherosclerotic plasma lipid profile and to have beneficial effects on insulin resistance and inflammation [23].

The heart's energy comes predominantly from FA oxidation (FAO), and PPAR*α* is expressed at a relatively high level in the heart. Moreover, PPAR*α* agonist treatment could induce the expression of various genes involved in FA utilization, including FA translocation, esterification, and *β*-oxidation, and reduce the expression of the genes involved in glucose uptake and use [24] (Table 1).

A relatively high-level expression of PPAR*α* also occurs in most cell types present in the vasculature, including ECs, VSMCs, monocytes, macrophages, and macrophage-rich regions of atherosclerotic regions [25]. In ECs, the activation of PPAR*α* could interfere with the metabolic processes involved in recruitment of inflammatory cells and regulation of redox responses, which would prevent vascular inflammation and injury [26, 27]. In addition, PPAR*α* agonists increase nitric oxide (NO) production in ECs and induce NO synthase (NOS) expression, suggesting a vasculoprotective effect [23] (Table 1).

Similar to other vascular cells, PPAR*α* has an anti-inflammatory effect in VSMCs. In addition, PPAR*α* activation can exert inhibition effects on VSMC proliferation and migration mediated by the nuclear factor-*κ*B, transforming growth factor-*β*/Smad, and the mitogen-activated protein kinase (MAPK) pathway. And the activation of PPAR*α* has proapoptotic effects on VSMCs by targeting the p38 MAPK signalling cascade [28] (Table 1).

In macrophages, activation of PPAR*α* exerts a vasculoprotective effect by attenuating TG accumulation and reducing tissue factor synthesis, matrix metallopeptidase-9, and tumour necrosis factor *α* (TNF-*α*) secretion [29]. Moreover, PPAR*α* helps to relive and stabilize the atherosclerotic plaques through promoting transport of cholesterol and enhancing collagen content [30, 31] (Table 1). PPAR*α* has also been shown to suppress platelet-activating receptor transcription in monocytes [32, 33]. Additionally, PPAR*α* activation could promote FAO, lipolysis, and adipocyte differentiation in adipocytes and improve insulin resistance without adipocyte lipid accumulation [34] (Table 1).

#### 1.2.2. PPAR*β*/*δ*

Accumulating evidence has shown the direct effects of PPAR*β*/*δ* on cardiovascular processes, such as endothelial function and angiogenesis [3]. PPAR*β*/*δ* is highly expressed in vasculature cell types including ECs, VSMCs, and monocyte-macrophages, and it contributes greatly to the function of these cell types [35, 36]. PPAR*β*/*δ* activation coordinates various functions in ECs, including the proliferation of ECs and endothelial progenitor cells, stimulating transcription of antioxidant enzymes, raising the phosphorylation of EC NOS (eNOS) and secretion NO, reducing inflammation and apoptosis, and regulating angiogenesis [37, 38] (Table 1). PPAR*β*/*δ* also regulates VSMC function through several mechanisms. PPAR*β*/*δ* represses VSMC proliferation, migration by sustentation of extra cellular matrix, suppression of apoptosis, and attenuation of senescence through increasing antioxidant enzyme genes and inhibiting inflammation [39, 40] (Table 1).

In macrophages, PPAR*β*/*δ* exerts an important role in the modulation of lipid/cholesterol metabolism and inflammatory responses [41]. Additionally, PPAR*β*/*δ* has vital effects on the regulation of brown or white adipose tissue FA transportation, oxidation metabolism, and thermogenesis [40, 42]. The evidence shows that the proper balance of PPAR*β*/*δ* activation is required to obtain beneficial effects on the outcome in ATH and chronic ischemic heart disease [37, 43, 44]. In the heart, PPAR*β*/*δ* is a crucial regulatory factor of primary myocardial FAO, and it is necessary to coordinate normal cardiac function and energy balance [45] (Table 1).

Usually, heart regeneration in the mammalian heart is limited to newborns [46]. However, Magadum et al. [47] demonstrated that the activation of PPAR*β*/*δ* by carbacyclin could promote cardiomyocyte proliferation. PPAR*β*/*δ* could also modulate the differentiation of adipocytes both independently and by targeting PPAR*γ*. This could regulate FA transportation, oxidation, and thermogenesis in white or brown adipose tissue and help to improve insulin sensitivity (Tanaka et al., 2017) (Table 1).

#### 1.2.3. PPAR*γ*

The cardioprotective effect of PPAR*γ* activation has been studied extensively, and it is considered to be a potential therapeutic target in CVDs [1]. PPAR*γ* is highly expressed in VSMCs, ECs, cardiomyocytes, macrophages, and adipocytes [48]. PPAR*γ* plays a crucial effect in inhibiting apoptosis and oxidative stress and improving endothelial function [49] (Table 1). PPAR*γ* plays a role against the inflammatory response of many cardiovascular cells, specifically ECs [50]. Activation of PPAR*γ* can decrease the expression of factors such as TNF-*α*, resistin, and interleukin 1. PPAR*γ* could also reduce inducible NOS increase and reactive oxygen species generation in macrophages. PPAR*γ* is a primary regulator of adipogenesis, and PPAR*γ* activation exerts important effects on the regulation of lipid metabolism in adipocytes and glucose homeostasis and adipogenesis in subcutaneous fat [51, 52] (Table 1).

Most studies have focused on the beneficial role of PPAR*γ* in preventing many cardiovascular disorders, such as insulin resistance [53, 54], ATH [55], hypertension, ischemia/reperfusion (I/R) injury [56], and dyslipidaemia [57]. In addition to regulating plasma lipoprotein concentrations, PPAR*γ* may modulate foam cell formation, affect the inflammatory response, and regulate plaque stability [58]. PPAR*γ* may also reduce the plasma level of atherogenic proteins. Recently, PPAR*γ* has been shown to exert neuroprotection in stroke both in rodent models and humans, which makes PPAR*γ* activators a potentially ideal treatment for ischemic brain injury [59, 60].

In the heart, PPAR*γ* protein expression was only at low to moderate levels. However, PPAR*γ* activation was demonstrated to cause cardiac dysfunction with significant changes in the metabolism of free FA and glucose. The therapeutic effects of PPAR*γ* ligands on cardiomyocytes are predominately attributed to their anti-inflammatory effects (Lee et al., 2015) (Table 1).

Above all, PPARs may have remarkable protective roles in various cell types involving in the pathophysiology of CVDs and could often be considered to be potential targets for therapeutic intervention of CVDs.

### 1.3. Crosstalk between MicroRNAs (miRNAs) and PPARS in CVDs

MiRNAs are defined as highly conserved endogenous noncoding RNAs, approximately 16–22 nucleotides in length [61]. MiRNAs function primarily by binding to 3′ untranslated regions (3′ UTR) of target miRNAs and usually decrease the expression of target genes at the posttranscriptional level [62–64]. MiRNAs are usually generated by a canonical pathway [65, 66]. The miRNA-loaded, RNA-induced silencing complex can silence the target gene expression by either targeting transcript degradation/decay or suppressing target transcript translation [13, 65].

According to the newly developed international genome feature nomenclature guidelines of miRNAs, many rules should be noted such as informing through the annotation without bias [67]. For instance, the unbiased “5p/3p” strand annotation is used to replace the “miR/miR^*∗*^” symbolism for deciphering the strand position from the pre-miRNA hairpin independently of its transcription status.

More than 2,000 miRNAs have been identified, and about 45,000 miRNA-targeting positions exist in the human genome, influencing the transcription of about 60% of genes [68]. MiRNAs can modulate almost all cellular functions and alterations of their expression/activity; this is observed in various pathological conditions, especially in CVDs [69–72].

Emerging evidence has suggested the miRNA/PPAR*γ* regulatory axis contributes greatly to CVDs and risk factors. Herein, the regulation of PPARs by miRNAs is reviewed in the context of PH, vascular dysfunction, heart disease, and obesity-associated cardiovascular risks. Reciprocal control of miRNAs expression by PPARs is also discussed. This will advance the understanding of the molecular pathway in CVDs and guide miRNA/PPAR axis-based therapeutic treatment in these diseases.

#### 1.3.1. Pulmonary Hypertension (PH)

It has been suggested that the miRNA/PPAR*γ* regulatory axis might contribute greatly to PH pathogenesis by regulating proliferation/apoptosis, communication, and normal function of PAECs and PASMCs.

**Figure 1:**
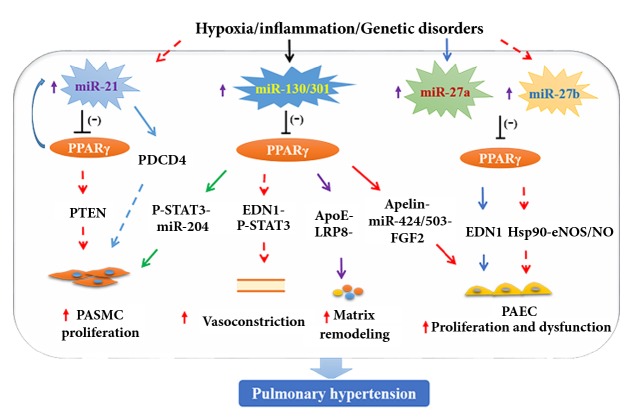
The regulation of peroxisome proliferator-activated receptors (PPARs) by microRNAs in pulmonary hypertension.* PASMC: pulmonary arterial smooth muscle cells; PAEC: pulmonary arterial endothelial cells; PTEN: phosphatase and tensin homolog deleted on chromosome 10; PDCD4: programmed cell death 4; STAT3: signal transducer and activator of transcription 3; EDN1: endothelin-1; APOE: apolipoprotein E; NO: nitric oxide; eNOS: EC nitric oxide synthase.*

The miR-130/301 family was demonstrated as a crucial regulator of PH by targeting PPAR*γ* (Figure 1) [73–75]. The miRNA-130/301 family was upregulated by various stimuli of PH in both animals and humans. It regulated proliferation and apoptosis of pulmonary vascular cells via inhibition of its target PPAR*γ*. The miRNA-130/301-PPAR*γ* axis modulated PAEC proliferation and apoptosis by regulating the signal pathway of apelin-miRNA-424/503-FGF2. For PASMCs, the miR-130/301-PPAR*γ* axis promoted cell proliferation through controlling miRNA-204 and signal transducer and activator of transcription 3 (STAT3) [75]. Furthermore, in murine models, chronic miR-130/301 treatment was necessary and sufficient to induce PH by targeting PPAR*γ* and the subordinated miRNA pathways [75]. The molecular crosstalk among various cell types was very important in the progress of PH. Bertero et al. [73] proved that the miR-130/301/PPAR*γ* axis in PAECs regulated various vasoactive factors, most significantly endothelin-1(EDN1), which serves as a vital regulator of vascular communication between PAECs and PASMCs, vasomotor tone, and PH manifestation* in vivo*. The miR-130/301-PPAR*γ* axis induced paracrine expression of STAT3 and promoted actinomyosin-dependent contraction of PASMCs by producing EDN1. In addition, the remodeling and stiffening of vascular extracellular matrix (ECM) were early and ordinary processes which aggravated PH. Moreover, Bertero et al. [74] found that miR-130/301 promoted ECM stiffening and further elevated YAP/TAZ through a feedback loop by targeting a PPAR*γ*-apolipoprotein E/LRP8 axis. And the suppression of microRNA-130/301 as well as apolipoprotein E by targeting PPAR*γ* signalling could ameliorate ECM remodeling and improve PH* in vivo *[74].

The dysfunction of PAECs was closely related to PH. The miRNA-27a/PPAR*γ* axis was confirmed to mediate mutually repressive actions in hypoxic human PAECs and also* in viv*o** (**Figure 1**) **[76]. Hypoxia, a common PH stimulus, increased the lungs' miRNA-27a expression and reduced PPAR*γ* levels, which stimulated increased EDN1 levels and proliferation of pulmonary vascular cells. However, activating PPAR*γ* by rosiglitazone (RSG) could directly reduce hypoxia-induced miRNA-27a transcription and then EDN1 levels, resulting in decreased PAECs proliferation [76].

In rats, miRNA-27b was highly expressed in PH, and inhibition of miRNA-27b attenuated monocrotaline-caused endothelial dysfunction and remodeling and then protected PH. Further, PPAR*γ* was demonstrated to directly modulate miRNA-27b expression in PAECs. Moreover, the miRNA-27a/PPAR*γ* axis regulated Hsp90-eNOS and NO signalling in human PAECs; this signalling was closely associated with the PH phenotype** (**Figure 1**) **[77].

MiRNA-21 was confirmed as an important regulator of PH pathogenesis by its promotion of PASMC proliferation. However, PPAR*γ* activation exerted an antiproliferative role through suppression of hypoxia-induced miRNA-21 transcription. Furthermore, RSG reduced miRNA-21 expression increased by hypoxic treatment both* in vitro* and* in vivo*, and it abolished decrease in phosphatase and tensin homolog deleted on chromosome 10 (PTEN) and PASMC proliferation [78]. The antiproliferative effects of RSG disappeared after PTEN depletion. In addition, miRNA-21 mimic reduced PTEN and promoted PASMC proliferation, while miRNA-21 suppression raised PTEN and reduced hypoxia-induced human PASMC proliferation** (**Figure 1**) **[78]. Green et al. found that the activation of PPAR*γ* could stimulate the central mediator of apoptosis programmed cell death 4 expression, by inhibiting miRNA-21 and promoting PASMC proliferation** (**Figure 1**) **[79]. These findings provided critical validation for developing the miRNA/PPAR*γ* axis-based therapeutics for PH.

#### 1.3.2. Vascular Dysfunction

**Figure 2:**
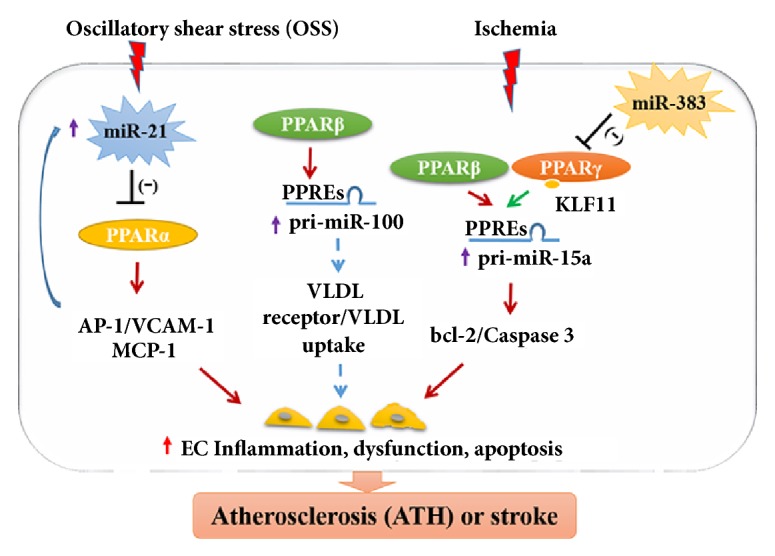
The regulatory function of microRNAs/peroxisome proliferator-activated receptors (PPARs) axis in the pathophysiology of atherosclerosis and stroke.* EC: endothelial cell; AP-1: transcription factor activator protein-1; VCAM-1: vascular cell adhesion molecule-1; MCP-1: monocyte chemotactic protein-1; VLDL: very low-density lipoprotein; KLF11: Kruppel like factor 11.*

Additionally, this miRNA/PPAR axis could provide novel therapeutic strategies for the treatment of ATH or stroke-related vascular dysfunction. Under conditions of endothelial dysfunction, adhesion of plasma monocytes to vascular ECs is a vital event contributing to vascular inflammation and further induces the development of ATH [80]. The crosstalk between miRNA-21 and PPAR*α* was shown to play key roles in proinflammatory molecule transcription and the adhesion of monocytes to ECs** (**Figure 2**) **[81]. The oscillatory shear stress induction of miRNA-21 inhibited PPAR*α* by directly targeting at the 3′ UTRs of PPAR*α*. Therefore, the repressive influence of PPAR*α* on the activation of transcription factor activator protein-1 was alleviated. And then, PPAR*α* inhibition promoted the transcription of adhesion molecules including vascular cell adhesion molecule-1 and monocyte chemotactic protein-1, finally resulting in EC inflammation** (**Figure 2**) **[81]. Moreover, the increase of miRNA-21 transcription further inhibited PPAR*α* expression from forming a positive feedback circuit.

As is well known, very low-density lipoprotein (VLDL) might enhance the permeability and apoptosis of ECs and increases inflammatory response, playing a crucial role in atherogenesis especially for coronary artery diseases [82]. In ECs, it was shown that the PPAR*β*/*δ* activation could inhibit VLDL receptor transcription and VLDL uptake through directly regulating miRNA-100, suggesting a vasculoprotective effect** (**Figure 2**)** [83].

Furthermore, the activation of PPAR*β*/*δ* or PPAR*γ* directly suppresses the transcriptional levels of proapoptotic miRNA-15a, playing a potential neuroprotective effect for ischemic stroke** (**Figure 2**) **[84]. The degeneration of cerebral vascular endothelial cells (CECs) is obviously related to blood-brain barrier breakdown as well as neuronal loss after cerebral ischemia. Upregulation of PPAR*β*/*δ* could alleviate oxygen glucose deprivation-activated miRNA-15a transcription in CECs. In addition, miRNA-15a could directly inhibit the translation of Bcl-2** (**Figure 2**)**. PPAR*β*/*δ* agonist obviously decreased ischemia-induced transcripts of miRNA-15a, promoted protein levels of Bcl-2, and reduced caspase-3 activity. Gain or loss of miRNA-15a function obviously reduced or increased OGD-activated CEC death, respectively [84]. Yin et al. further demonstrated that KLF11, a novel PPAR*γ* coregulator, interacted with PPAR*γ* and suppressed miRNA-15a, leading to endothelial protection both in CEC cultures and cerebral microvasculature following ischemic stimuli [85]. Additionally, miRNA-383 could contribute to focal cerebral ischemia through modulating PPAR*γ* transcription at the posttranscriptional level* in vitro* and* in vivo *[86]. MiRNA-383 and PPAR*γ* might serve as potential therapeutic strategies for stroke.

#### 1.3.3. Heart Diseases

**Figure 3:**
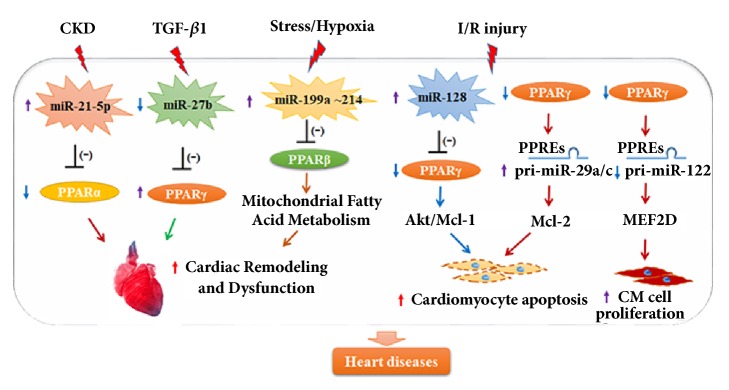
The regulatory function of microRNAs/peroxisome proliferator-activated receptors (PPARs) axis in cardiomyocyte apoptosis, and cardiac remodeling and dysfunction.* CKD: chronic kidney disease; TGF-β1: transforming growth factor β1; I/R: ischemia/reperfusion; Akt: serine/threonine kinase 1; Mcl-1: myeloid leukaemia cell differentiation protein-1; Mcl-2: an anti-apoptotic Bcl-2 family member; MEF2D: myocyte enhancer factor 2D. CM: cardiac myxoma.*

CVDs caused the leading death in patients with chronic kidney disease [87, 88]. PPAR*α*, a primary PPAR isoform in the heart, was shown to be a direct target of miRNA-21-5p** (**Figure 3**)**. Suppression of miRNA-21-5p could alter gene expression in PPAR*α* modulated pathways in the left ventricle. Moreover, cardiac function and left ventricle dilation were improved by therapeutic delivery of low-dose PPAR*α* agonist in rats with 5/6 nephrectomy [89].

Cardiac injury is associated with marked induction of TGF-*β*, and the miRNA-27b expression was shown to be suppressed by TGF-*β*1* in vitro.* MiRNA-27b overexpression was sufficient to induce cardiac hypertrophy and dysfunction* in vitro *and* in vivo*. Furthermore, PPAR*γ* was confirmed as a direct target of miRNA-27b in cardiomyocytes** (**Figure 3**) **[90].

*In vitro*, miRNA-128 suppression enhanced the activation of Akt (phosphorylated [p]-Akt), myeloid leukaemia cell differentiation protein-1 (Mcl-1), and PPAR*γ* expression in the myocardium. MiRNA-128 suppression further attenuated cardiomyocyte apoptosis induced by myocardial I/R injury. The effects induced by miRNA-128 inhibition could be improved by the directly targeted activation of PPAR*γ *** (**Figure 3**) **[91].

Pioglitazone (PIO), a PPAR*γ* agonist, has been shown to prevent myocardial I/R injury. PIO treatment could directly decrease miRNA-29a and miRNA-29c agonist expression levels and promote the transcription of Mcl-2, an antiapoptotic Bcl-2 family member** (**Figure 3**) **[92]. Thus, inhibition of miRNA-29 by targeting enhanced PPAR*γ* showed an antiapoptotic effect, which protected the heart against I/R injury [92].

Several studies showed that abnormalities in energy production and cardiac lipid homeostasis were closely related to HF. Under hemodynamic stress, PPAR*β*/*δ*, the crucial regulator of the energy metabolism of the heart, could be directly regulated by cardiac hypoxia that activates the microRNA cluster miRNA-199a-214. Moreover, miRNA-214 silencing enhanced cardiac contractility and improved mitochondrial FAO by inhibiting PPAR*β*/*δ *** (**Figure 3**) **[93].

Furthermore, the PPAR/miRNA axis was shown to be a crucial regulator of cardiac myxoma (CM), a predominant cardiac tumour especially in the young [94]. Activation of PPAR*γ* could directly regulate miRNA-122 by binding to the PPRE in the promoter region and inhibit myocyte enhancer factor 2D (MEF2D) expression, an important biomarker of CM** (**Figure 3**)** [95]. Further experiments demonstrated that the decrease of MEF2D regulated by miR-122/PPAR*γ* axis inhibited CM cell proliferation. These results suggest that the PPAR*γ*/miRNA-122 signalling pathway might serve as a novel target to treat CM.

#### 1.3.4. Obesity-Associated Cardiovascular Risks

**Figure 4:**
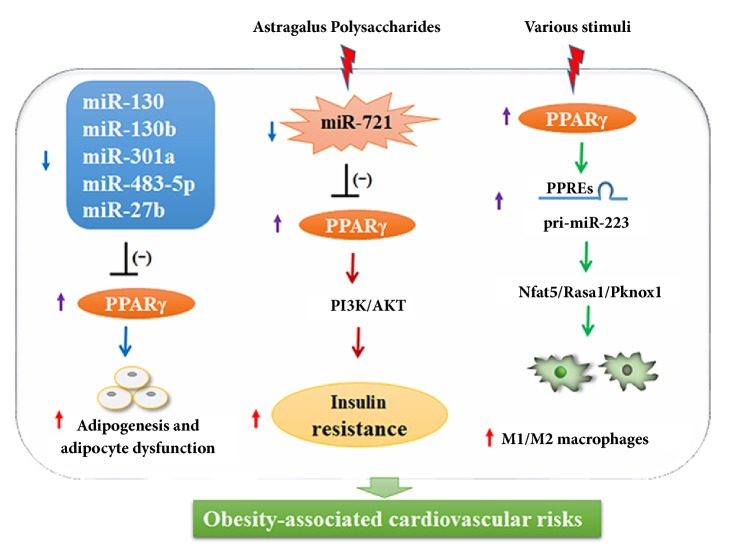
The regulatory function of microRNAs/peroxisome proliferator-activated receptors (PPARs) axis in obesity-associated cardiovascular risks.* PI3K: phosphatidylinositol-4,5-bisphosphate 3-kinase; Akt: serine/threonine kinase 1; Nfat5: nuclear factor of activated T-cells 5; Rasa1: RAS p21 protein activator 1; Pknox1: Pbx/knotted 1 homeobox 1; M1/M2 macrophage: proinflammatory M1 macrophage /anti-inflammatory M2 macrophage.*

Several miRNAs play vital roles in antiobesity by suppressing the expression of PPAR*γ*, which regulates lipogenesis and adipogenesis. MiRNA-130 has been shown to reduce adipogenesis by repressing PPAR*γ *** (**Figure 4**) **[96]. In addition, the fat deposition in recipient primary-cultured porcine adipocytes could be decreased by microvesicle-shuttled miRNA-130b through inhibiting PPAR*γ* [97]. In a preadipocyte cell line 3T3-L1, miRNA-301a could modulate adipocyte dysfunction via directly suppressing PPAR*γ* during obesity-related inflammation [98]. Procyanidins could promote lipolysis on adipose metabolism; grape seed procyanidin B2 was shown to inhibit adipogenesis in 3T3-L1 cells by regulating the miRNA-483-5p/PPAR*γ* signalling pathway [99]. A Chinese medicine, Astragalus polysaccharides, could attenuate TNF*α*-induced insulin resistance by inhibiting miRNA-721 expression, activating PPAR*γ*, and enhancing PI3K/Akt signalling in 3T3-L1 adipocytes** (**Figure 4**) **[100]. MiRNA-27b was shown to exert an antiadipogenic effect on human multipotent adipose-derived stem cells by directly inhibiting PPAR*γ* and another regulator of adipogenesis, C/EBP*α*, at early onset of adipogenesis. Further, overexpression of miRNA-27b could suppress expression of the adipogenic marker gene and TG accumulation during the late stages of adipogenesis [101].

Polarized activation of adipose tissue macrophages is crucial to maintain normal adipose tissue function and mediate obesity-associated cardiovascular risk and metabolic abnormalities [102]. The PPAR*γ*/miRNA-223 regulatory axis modulated macrophage polarization by regulating expression of distinct downstream genes under various stimuli** (**Figure 4**) **[103]. In BM-derived macrophages, miRNA-223 transcription was directly promoted by PPAR*γ* under Th2 stimuli, and Rasa1 (a member of the nuclear factors of the activated T-cells family of transcription factors), Nfat5 (a member of the GAP1 family of GTPase-activating proteins), and the proinflammatory regulator Pknox1 were identified as miRNA-223 targets. These findings further supported the theory that the mutual regulation of miRNAs and PPAR signalling might be a novel target for alleviating obesity-associated cardiovascular risks.

## 2. Conclusion

PPARs have a wide spectrum of biological activities relevant to the prevention and treatment of CVDs, including regulating energy homeostasis, promoting proliferation, and inhibiting inflammation, oxidative stress, and apoptosis in vascular cells, cardiomyocytes, and adipocytes. Increasing evidence suggests that miRNAs serve as key mediators of pathogenesis in CVDs and their risks. However, the important and versatile regulatory function of the interaction of PPARs and miRNAs has not been studied extensively. This review stated that PPARs can be inhibited through posttranscriptional mechanisms that involve miRNAs in the progress of PH, vascular dysfunction, heart diseases, and obesity-related CVD risks. In addition, the ability of PPARs to change the posttranscriptional expression of target miRNAs through miRNA signalling in the CVD pathophysiology was also discussed. This indicates that strategies targeting PPAR can regulate not only transcriptional but also posttranscriptional regulation of cardiac and vasoactive mediators to favourably modulate CVD pathogenesis. In conclusion, the review illustrated a direct link between miRNAs and nuclear receptor PPARs in the context of PH, vascular dysfunction, heart diseases, and obesity-related cardiovascular risks and demonstrated that targeting the miRNA/PPAR axis may represent a novel therapeutic approach for CVDs.

## Figures and Tables

**Table 1:** The beneficial effects of peroxisome proliferator-activated receptors (PPARs) activation on cardiovascular diseases and risks.

Diseases	Cell	PPAR*α*	PPAR*β*/*δ*	PPAR*γ*
**Vessel wall**	**Endothelial cell**	**Nitric oxide release↑** **Inflammation↓**	**Nitric oxide release↑** **Proliferation ↑** **Apoptosis ↓** **Inflammation ↓**	**Apoptosis ↓** **Reactive oxygen species ↓** **Inflammation ↓**
	**Vascular smooth muscle cell**	**Apoptosis↑** **Proliferation↓** **Migration↓** **Inflammation↓**	**Proliferation ↓** **Migration ↓** **Apoptosis ↓**	**Proliferation ↓** **Migration ↓** **Apoptosis ↓**
	**Monocyte/** **Macrophage**	**Inflammation↓** **Lipid accumulation↓** **Reverse cholesterol transport ↑**	**Lipid/cholesterol** **metabolism ↑** **Inflammation ↓**	**Reactive oxygen species ↓** **Nitric oxide synthase ↑** **Reverse cholesterol transport ↑**

**Heart **	**Cardiomyocyte**	**Fatty acid metabolism ↑** **Glucose uptake and oxidation ↓**	**Proliferation ↑** **Energy balance ↑** **Metabolic modulation ↑**	**Inflammation ↓**

**Risk factors**	**Adipocyte**	**Adipocyte differentiation ↑** **Lipolysis ↑** **Fatty acid oxidation ↑**	**Adipocyte differentiation ↑** **Fatty acid metabolism ↑**	**Adipocyte differentiation ↑** **Lipolysis↑**
